# Results from a Community-Based Smoking Cessation Treatment Program for LGBT Smokers

**DOI:** 10.1155/2013/984508

**Published:** 2013-06-12

**Authors:** Alicia K. Matthews, Chien-Ching Li, Lisa M. Kuhns, Timothy B. Tasker, John A. Cesario

**Affiliations:** ^1^University of Illinois at Chicago, College of Nursing, 845 South Damen Avenue, Chicago, IL 60612, USA; ^2^Rush University Medical Center, College of Health Sciences, Department of Health Systems Management, 1635 West Congress Parkway, Chicago, IL 60612, USA; ^3^Ann & Robert H. Lurie Children's Hospital of Chicago & Northwestern University Feinberg School of Medicine, 225 East Chicago Avenue, Chicago, IL 60611, USA; ^4^University of Illinois at Chicago, Department of Psychology, 1007 West Harrison Street, Chicago, IL 60607, USA

## Abstract

*Introduction*. Little is known about lesbian, gay, bisexual, and transgender (LGBT) people's response to smoking cessation interventions. This descriptive study examined the benefits of a community-based, culturally tailored smoking cessation treatment program for LGBT smokers. *Methods*. A total of *N* = 198 LGBT individuals recruited from clinical practice and community outreach participated in group-based treatment. Sessions were based on the American Lung Association's “Freedom from Smoking Program” (ALA-FFS) and were tailored to LGBT smokers' needs. Seven-day smoking point prevalence abstinence served as the primary outcome. *Results*. Participants (M age = 40.5) were mostly White (70.4%) and male (60.5%) and had at least a college degree (58.4%). Forty-four percent scored in the moderate range on the Fagerström Test for Nicotine Dependence pretreatment, and 42.4% completed treatment (≥75% sessions). Higher educational attainment and use of nicotine replacement therapy (NRT) were associated with treatment completion. Self-reported quit rates were 32.3% at posttreatment assessment. Treatment attendance (OR = 2.45), use of NRT (OR = 4.24), and lower nicotine dependency (OR = 0.73) were positively associated with quitting smoking. *Conclusions*. Results suggest the benefits of offering LGBT smokers culturally tailored smoking cessation treatments. Future research could improve outcomes by encouraging treatment attendance and promoting NRT uptake.

## 1. Introduction

Members of the lesbian, gay, bisexual, and transgender (LGBT) communities have been identified as a population group at elevated risk for smoking-related health disparities [1, 2]. For example, results from national samples of LGBT persons consistently demonstrate disparate rates of tobacco use, with prevalence rates of smoking roughly twice those of heterosexuals [2–5]. A recent study found that tobacco use contributes to the greater than expected rates of acute respiratory illnesses among LGBT young adults compared to a heterosexual comparison group [1]. Furthermore, LGBT persons have higher than expected prevalence of risk factors (e.g., heavy drinking) for diseases associated with or exacerbated by smoking (e.g., heart disease, HIV infection, and lung cancer) [6–8]. As such, tobacco prevention and control have emerged as a significant priority for researchers, policy makers, and national organizations focused on LGBT concerns. Yet, best practices for smoking cessation among LGBT populations are glaringly absent because of the lack of empirical research [9, 10]. Testing innovations developed by community organizations, local health departments, and others outside of academic centers (i.e., practice-based research) can help address such gaps [11, 12].

In the general population the majority of current smokers (68.8%) report wanting to quit, and more than half of smokers (52.4%) have made a quit attempt in the previous year [13]. Similarly, members of the LGBT communities are knowledgeable about the risks associated with smoking and interested in quitting smoking. For example, awareness of the health risks associated with smoking and acceptance of tobacco control activities was high in a recent study of smoking-related knowledge, attitudes, and behaviors of LGBT persons [14]. In addition, high levels of awareness of smoking prevention campaigns, stated intentions to quit smoking, and reports of recent quit attempts have recently been observed among LGBT samples [15, 16]. With regard to treatment seeking, data collected from a large (*N* = 1633) convenience sample of current LGBT smokers suggested that the majority (75%) were willing to talk with their providers about access to smoking cessation treatments [17]. About one-quarter of those respondents indicated their intention to use nicotine replacement therapy (NRT) during their next quit attempt [17], a rate consistent with the general population of smokers [18]. Taken together, these findings suggest both a receptivity to and an opportunity for further engaging LGBT smokers in smoking cessation efforts.

Increasing the availability of evidence-based treatments to LGBT smokers is central to reducing the high rates of tobacco use in this group [19]. Although effective smoking cessation treatments are now widely available [9], the majority of these treatments were designed as interventions for the general population and not culturally tailored to the specific needs of any particular subgroup. A large body of evidence suggests that psychosocial and cultural factors related to smoking cessation are differentially distributed among subgroups and that addressing these differences may improve treatment outcomes [9]. Indeed, a survey conducted with gay/bisexual male smokers revealed a stronger preference for smoking cessation programs tailored to gay men over generic programs [20]. Culturally tailored interventions have been shown to be effective in improving outcomes for a range of health promotion interventions [21, 22]. Examples of tailored smoking cessation interventions include programs for people with chronic illnesses such as cancer [23]; racial and ethnic minority youth [24]; and members of Hispanic [25], African American [26], and Indigenous populations [27]. As members of a highly stigmatized minority group, LGBT persons experience unique and formidable barriers to smoking cessation including stress associated with discrimination, internalized homophobia, and limited access to culturally competent health care [28]. To address these unique concerns, efforts have been made to develop culturally competent and tailored smoking cessation treatment materials that address the cultural and psychosocial needs of LGBT smokers [29–32].

Despite the availability of culturally specific smoking cessation materials, little research exists on outcomes for LGBT smokers based on either tailored or nontailored treatment programs [2, 9, 33]. One example is provided by Harding and colleagues [34] who conducted a pilot smoking-cessation intervention that was culturally tailored for gay men in the United Kingdom (*N* = 69). Their intervention consisted of four weekly two-hour sessions together with NRT. Outcomes were promising with biochemically verified quit rates one month after treatment at 48%. Walls and Wisneski [35] reported on a preliminary evaluation of the acceptability of a set of community-based smoking cessation classes, “The Last Drag,” that were culturally tailored to the sociocultural needs of LGBT smokers (*N* = 44). The smoking cessation classes were based on a culturally adapted version of the American Lung Association's Freedom from Smoking (ALA-FFS) curriculum [36] and included seven group-based counseling and peer support sessions. Of the 44 individuals who participated, 36 were in attendance in the final class, and 89% of those self-reported being quit. Ratings of the cultural appropriateness of the program were also strong. In a larger scale evaluation of “The Last Drag” program, Eliason and colleagues [19] reported results from all participants who completed one or more sessions from August 2005 to January 2010 (*N* = 233). Self-reported quit rates for the sample were 53% at one month and 36% at 3 and 6 months, respectively. Finally, a recent study examined smoking cessation outcomes based on sexual orientation following a nontailored smoking cessation intervention [37]. A total of 54 sexual minority men (SMM) and 243 heterosexual male smokers received 8 weeks of treatment that included nicotine patch, Bupropion, and individual behavioral counseling sessions. Rates of quitting among SMM were nearly identical to those of heterosexual males (week 8; SMM = 59% versus heterosexual = 57%). The results from the few existing smoking cessation treatment programs involving LGBT smokers are promising; however, additional research is needed to establish culturally appropriate and evidence-based treatment strategies. In the absence of extensive research-based evidence, data available from existing smoking cessation treatments offered as part of routine clinical services can provide important information about smoking cessation treatments in “real world settings” and can guide future research and provide models for other community-based treatment centers.

## 2. Specific Aims

 The purpose of this study was to evaluate the treatment outcomes associated with a culturally tailored smoking cessation treatment program for LGBT smokers that were offered as part of clinical practice in a community-based health center. We sought to answer the following research questions: (1) What were the participation rates of LGBT smokers taking part in a community-based smoking cessation treatment program? (2) Was there evidence that the culturally tailored programs were effective for reducing smoking behaviors? (3) What participant characteristics (demographic, smoking, and treatment attendance) were associated with better treatment outcomes? 

## 3. Methods

### 3.1. Sample

 Data for this study were obtained from a series of smoking cessation treatment programs tailored to LGBT smokers and offered at Howard Brown Health Center (HBHC) from 2002 to 2006. The original program, “Call It Quits,” was developed as part of a research grant awarded to HBHC. After completion of the research grant, additional funding was obtained to offer smoking cessation treatments as part of clinical services at HBHC. A total of three separate programs were offered including “Call It Quits” (CIQ, 2002, a total of 105 smokers participated in 15 cessation groups), “Bitch To Quit” (BTQ, 2005-2006, a total of 33 smokers participated in 5 cessation groups), and “Put It Out” (PIO, 2006–2008, a total of 60 smokers participated in 10 cessation groups). Each of these clinical programs were based on the core CIQ curriculum; however, some variations in approaches occurred, including changes in program names, assessment measures, the inclusion of a peer support “buddy,” or the number of treatment sessions offered.

 The smoking cessation classes were advertised using a range of strategies including provider referrals and information posted in HBHC's medical clinic and through postings and materials (e.g., posters, flyers, and matchbooks) in community venues. When possible, additional funding was obtained to do specific outreach and advertising to subpopulations of the LGBT communities based on race/ethnicity, gender, or gender identity. These groups followed the same treatment procedures as the standard groups but were held in locations specifically serving the needs of members from these communities. In general, eligibility criteria for the smoking cessation treatment programs included (1) self-identified as LGBT, (2) age 18–65, and (3) current cigarette smoker (any level of smoking). Depending on when smoking cessation services were received, treatment was either free of charge or provided on a sliding scale fee schedule according to income. No interested smokers were denied treatment because of inability to pay. Data from each of the treatment groups were combined for statistical analyses. The Institutional Review Board of HBHC has approved the secondary analysis of programmatic data. 

#### 3.1.1. Treatment Approach

All treatment programs were developed based on the well-established ALA-FFS, a group-based and cost-effective group smoking cessation treatment [36, 38]. The underlying premise of the ALA-FFS cognitive/behavioral program is that smoking is a learned habit. The program focuses on how to make behavioral changes and to quit smoking in the context of a group environment. The ALA-FFS program was selected because it incorporates many of the best practices identified by the Treatment Practice Guidelines [39] including skill building and preparation for quitting, intratreatment social support, and optimal treatment dosing (i.e., 4–7 sessions). 

Because the ALA-FFS program was designed for the mainstream USA population, the program was adapted for the LGBT community. The culturally tailored smoking cessation program maintained the core cognitive and behavioral approaches and smoking education content used in the standard ALA-FFS program. The goal of the tailoring was to adapt the existing ALA-FFS cessation materials to the culture, norms, and beliefs of LGBT men and women and to address the unique factors associated with their smoking behaviors. First we focused on strategies to increase the level of trust and acceptability of the program. These included hosting all smoking cessation treatments at LGBT community organizations, employing LGBT-identified smoking cessation counselors, and branding recruitment and program materials with LGBT-specific images. Next we included culturally specific content into treatment materials. The culturally specific content was informed by information obtained from focus group interviews with LGBT smokers that explored barriers and facilitators to smoking cessation (*N* = 9 groups) [40], published guidelines for conducting culturally competent smoking cessation treatments [41], a panel of LGBT health experts, a review of the literature, and recommendations from existing tailored smoking cessation programs (e.g., QueerTIPs [30]). A few examples of culturally tailored elements included a discussion of health concerns for LGBT smokers (e.g., HIV/AIDS, hormone use among transgender smokers); the role of smoking in the LGBT culture; stress due to homophobia as triggers for smoking and relapse; ways to increase social support for nonsmoking; relationships between bar culture, drinking, and smoking; targeting of the LGBT communities by the tobacco industry; providing smoking statistics/rates specially for the LGBT communities; and the inclusion of themes related to community empowerment and health. The combined information was folded into the ALA-FFS curriculum to create the tailored “Call It Quits” (later renamed as “Bitch To Quit” and “Put It Out”) smoking cessation program materials and the training curriculum for group facilitators. 

A total of 30 separate treatment groups were offered. The majority of the smoking cessation groups were conducted at the HBHC. Additional funding was obtained to offer CIQ at three other community-based locations serving the needs of LGBT smokers from diverse ethnic and racial backgrounds. LGBT-identified professional facilitators conducted each group session. In the CIQ sessions only, LGBT ex-smokers served as peer support “buddies.” Smoking cessation counselors were trained through the American Lung Association of Metropolitan Chicago (ALAMC). Treatment groups were offered once weekly and ran for approximately 90 minutes each. The number of weekly treatment sessions varied somewhat across the differing treatment groups (CIQ = 8 sessions, BTQ = 8 sessions, PIO = 6 sessions). Outcome and process measures were collected at baseline, weekly, and at the end of treatment (approximately one month after quit date).

#### 3.1.2. Nicotine Replacement

NRT (i.e., patches) was provided free of charge as part of the PIO sessions from 2006 to 2008. Use of nicotine replacement and other pharmacotherapies during the course of treatment was measured for all treatment participants regardless of whether NRT was offered as part of treatment. 

### 3.2. Measures

#### 3.2.1. Participant Background Characteristics

Demographic characteristics measured as part of the baseline assessment included age, race (Asian, Black, White, other), gender (male, female, transgender), education (high school or lower, some college, college and above), employment status (full time, part time, unemployed, retired, disabled, other), annual income ($0–10,999, $11–20,999, $21–30,999, $31–49,999, $50,000 and over), and sexual orientation (gay, lesbian, bisexual, other). 

#### 3.2.2. Smoking Behaviors and Attitudes

Nicotine dependence was measured by the Fagerström Test for Nicotine Dependence [42]. This test assesses a smoker's level of dependency on nicotine and has demonstrated validity and reliability in previous research. Scores range from 0 to 10 with higher scores suggesting more nicotine dependence. Nicotine dependence was classified into three levels based on these composite scores: low (0–3), medium (4–6), and high (7–10). Smoking frequency was asked by the following single item: “*How many cigarettes/day do you smoke?*” (31–40 or more cigarettes, 21–30 cigarettes, 11–20 cigarettes, 10 or less cigarettes). Attitudes towards quitting were measured at baseline using four individual questions developed to measure cognitive factors associated with quitting behavior: (1) “*How much do you want to quit smoking?*” (definitely not, probably not, not sure, probably yes, definitely yes); (2) “*How important is it for you to quit smoking?*” (not at all, a little, moderately, very, extremely); (3) “*Do you intend to quit smoking?*” (definitely not, probably not, not sure, probably yes, definitely yes); and (4) “*How confident are you in your ability to quit smoking completely?*” (not at all, a little, moderately, very, extremely). These items were all worded in the same direction such that higher scores on each corresponded to more agreement with the question. 

#### 3.2.3. Treatment Outcomes

The primary outcome measured was self-reported seven-day point prevalence abstinence measured at completion of the smoking cessation intervention sessions. Individuals who had missing data for quit-smoking status were treated as nonquit based on an intention to-treat (ITT) approach to data analysis. Session attendance, a measure of treatment dose, was also measured as an important treatment outcome. Given that the number of treatment sessions differed somewhat between treatment programs (CIQ = 8 sessions, BTQ = 8 sessions, and PIO = 6 sessions), treatment completion was operationally defined as attending 75% or more of all available treatment sessions. Finally, all participants were asked whether they used any kind of stop-smoking medications during treatment, including nicotine replacement (e.g., patch, gum) or medications (e.g., Chantix, Bupropion).

### 3.3. Data Analysis

Data were available from *N* = 198 LGBT smokers who completed at least one session of the culturally tailored smoking cessation programs. Descriptive statistics were computed for all variables. Comparisons of characteristics between smoking cession groups (i.e., CIQ, BTQ, and PIO) were performed using Chi-Square (*χ* ^2^) tests for categorical variables and Analysis of Variance (ANOVA) for continuous variables. Multivariate binary logistic regression was performed to describe associations between smoking status and demographic characteristics, smoking attitudes, smoking characteristics, and treatment factors (i.e., use of NRT and number of sessions completed). Finally, additional multivariate linear regression analyses were conducted to examine the associations between session attendance and demographic characteristics, smoking attitudes, smoking characteristics, and treatment factors. All analyses were performed in SAS version 9.2.

## 4. Results

### 4.1. Demographic Characteristics

The mean age of the participants was 40.48 years (SD = 9.22). The majority of participants were White (70.4%) and male (60.5%) and had at least a college degree (58.4%). As shown in Table 1, the participants in each of the three smoking cessation treatment programs differed significantly based on demographic characteristics. CIQ had the highest proportion of male (76.7%) and unemployed people (43.3%); BTQ had the highest proportion of non-White (39.4%), female (93.9%), employed people (75.8%), and annual income more than $50,000 (45.5%); and PIO had the highest proportion of White people (83.3%), with these differences reaching statistical significance. Accordingly, a set of key demographic factors was used as control variables in subsequent analyses.

### 4.2. Smoking Behaviors and Smoking Cession Outcomes

At pretreatment, the majority of program participants reported definitely wanting to (62.4%) and intending to quit smoking (57.2%), and the vast majority of all participants indicated that it was either very or extremely important to quit smoking (85.1%). However, the proportion reporting that they were either very or extremely confident in their ability to quit smoking was comparatively lower (43.3%). The highest proportion of participants smoked 11–20 cigarettes per day (39.9%). Another 35.9% were light smokers (0–10 cigarettes per day) and 24.3% were heavy smokers (21+ cigarettes per day). Based on the Fagerström measure, mean nicotine dependency scores for all participants indicated moderate levels of dependency (*M* = 4.18, SD = 2.49). Nearly one-fifth of the sample (17.9%) scored in the high nicotine dependence range on the Fagerström measure. Significant differences in smoking characteristics were also observed for participants in each of the three different treatment groups (see Table 1). Treatment attendance rates were modest with 42.4% of participants completing ≥75% of sessions. Overall 32.3% of all participants self-reported having quit at the end of treatment. 

### 4.3. Multivariate Predictors of Treatment Outcomes

Multivariate analyses were conducted to assess the association between quit-smoking status and smoking characteristics (nicotine dependence), attitudes about quitting (want to quit, important to quit, intend to quit, confidence to quit), and treatment factors (session attendance and use of stop-smoking medications) controlling for demographic characteristics (race, gender, education, sexual orientation). Results showed that baseline levels of nicotine dependence, use of any stop-smoking medications as an adjunct to group counseling, and number of sessions attended were significantly associated with quit-smoking status. Any use of nicotine replacement (OR = 4.24, 95% CI = 1.23–14.50) and increased number of sessions attended (OR = 2.45, 95% CI = 1.45–4.15) were associated with higher odds of quitting smoking, respectively. However, increased nicotine dependence scores were associated with lower odds of quitting smoking (OR = 0.73, 95% CI = 0.57–0.94) (see Table 2).

Multivariate analyses were also performed to examine the associations between session attendance rates and smoking characteristics (nicotine dependence), attitudes about quitting (want to quit, important to quit, intend to quit, confidence to quit), and treatment factors (nicotine replacement use) while controlling for each demographic characteristic (race, gender, education, sexual orientation). After introducing all demographic and smoking characteristics in the multivariate model, education and nicotine replacement use still significantly associated with session attendance. Participants with some college education or less had lower session attendance rates compared to those with a college degree or more education (*β* = −0.10, *p* < 0.05). People who had ever used nicotine replacement had higher session attendance rates than those who had not used it (*β* = 0.10, *p* < 0.05) (see Table 3).

## 5. Discussion 

Scant research exists on effective smoking cessation treatments for LGBT smokers. Evaluation of smoking cessation treatments offered as part of clinical or behavioral health services provides opportunities to answer important questions about LGBT smoking cessation and can help establish the evidence base for treating tobacco addiction in LGBT smokers. The purpose of this paper was to address a significant gap in the literature on tobacco cessation by reporting on the outcomes associated with culturally tailored treatment programs for LGBT smokers. Study results suggest that culturally specific smoking cessation treatments offered in a community setting are beneficial for reducing smoking among LGBT smokers. Contrary to data suggesting low awareness and engagement of LGBT smokers with formal treatment programs [16], we demonstrated the ability to enroll and retain LGBT smokers into a community-based and intensive group smoking cessation program. Further, quit rates were on average consistent with outcomes associated with general population results from the ALA-FFS program [36]. These promising findings warrant future research to replicate results and to examine differences in smoking cessation outcomes from the tailored and nontailored ALA-FFS programs. 

### 5.1. Quit Rates and Factors Associated with Quitting

Overall quit rates were in the expected range for community-based smoking cessation treatments and ranged from 23.3 to 39.1% at the end of treatment. These intent-to-treat, quit rates at end of treatment and followup are comparable to those observed in mainly Caucasian smokers engaged in similar treatment (i.e., with nicotine patch and intensive counseling) [9]. However, the same contextual factors observed in LGBT communities that hinder smoking cessation (e.g., stress associated with discrimination, fewer deterrents to smoking, and limited access to culturally competent health care) [28] may also serve as formidable barriers to long-term cessation and should be systematically addressed as part of intensive relapse prevention treatment. As such, additional research is needed to examine maintenance of quit rates over time. 

In the general population, quit rates following smoking cessation treatment vary substantially based on demographic characteristics such as race/ethnicity. However, in multivariate analyses, demographic characteristics were not statistically significant predictors of the quit status of program participants. Instead, smoking- and treatment-related variables had direct effects on quit outcomes. The likelihood of being a nonsmoker was positively associated with use of stop-smoking medications and session attendance rates such that the odds of quitting were more than doubled with the use of any medications and completion of more treatment sessions. It should be noted that, in general, the use of stop-smoking medications was relatively low across program participants (24.2%). In general, nicotine dependency scores were in the moderate range and were associated with quit status such that those smokers with higher levels of nicotine dependency had lower odds of being quit after treatment. The relationship between nicotine dependence and lower quit rates suggests the need for increasing awareness and uptake of pharmacological approaches as key adjuncts to stop-smoking counseling programs.

Treatment completion rates were modest at 42.4% (range 31.7%–54.6%). After introducing all demographic and smoking characteristics in the multivariate model, education level and nicotine replacement use were significantly and positively associated with session attendance rates. Given the association between treatment attendance and quit outcomes, future initiatives should focus on strategies increasing adherence to treatment. 

### 5.2. Improving LGBT Smoking Cessation Interventions

A strength of the smoking cessation treatments offered at HBHC was the adaption of an existing and evidence-based program. This approach was consistent with calls to increase access to treatments for priority populations by disseminating effective behavioral interventions [11]. Other strategies to increase the impact and cultural competency of smoking cessation treatments have been well described [41] and minimally include the following: (1) conduct the treatment programs in settings that are considered safe to community members; (2) treatments should be administered by facilitators with expertise in smoking cessation and cultural competency in working with the LGBT communities; (3) distribute appropriate, inclusive, and creative promotional materials through community-based outlets; (4) provide cultural competency training to all non-LGBT staff who interact with participants; and (5) modify the curricula to include LGBT-specific and other culturally relevant information for participants. In addition, our findings suggest that the core cognitive and behavioral components of the ALA-FFS curriculum are beneficial for reducing smoking behaviors. Consistent with treatment and practice guidelines and our findings, it is important to include stop-smoking medications as a standard part of treatment. That being said, adherence to NRT was low among participants even when it was offered free of charge. As such, additional emphasis should be placed on identifying and addressing barriers to uptake of NRT as a part of standard treatment.

 In addition, focus groups were used to tailor these treatment programs to the needs of LGBT smokers. Several years have passed since this qualitative research was conducted and the findings may no longer be applicable to the current smokers based on a number of factors including identity factors (e.g., queer, gender nonconforming), smoking behaviors (e.g., menthol, light smokers, use of new tobacco products), barriers and facilitators to smoking cessation, and types of images and messaging that are appropriate. Consequently, additional formative research may be needed to revise and refine materials to address the needs of current LGBT smokers. 

### 5.3. Strengths and Limitations

The tailored curriculum retained the essential effective components of the ALA-FFS program, only deviating from the standard intervention in minimal ways. Further, facilitators were trained by the ALAMC. As such, we believe we were able to maintain the integrity of the program and that the adapted intervention was largely equivalent to the standard ALA-FFS program. Additional strengths of the study included culturally tailoring an existing and effective smoking cessation treatment program for LGBT smokers, measurement of demographic and smoking variables that may impact treatment outcomes, and posttreatment follow-up of treatment participants. However, the results should be considered in light of the study limitations. For example, the majority of these quit rates were not biochemically confirmed by objective measures and there was no comparison control condition. As such, these initial promising findings need replication and extension using more robust methodology. Additionally, we did not collect any culturally specific measures (e.g., minority stress) that may have provided additional insight into factors that may influence within-group differences in smoking behavior. Although basic demographic and smoking variables were measured as part of the treatment programs, a full battery of smoking questions was not included. Finally, no data were available on longer-term quit rates associated with the program. 

## 6. Conclusions

Study findings demonstrate the feasibility and benefits of providing smoking cessation treatments for LGBT smokers as part of clinical services. Future studies should build on the success of this clinical program of smoking cessation treatment by systematically evaluating the benefits of a culturally tailored intervention for improving smoking cessation outcomes in LGBT smokers. A research program should address the limitations of our clinical program by (1) improving the internal validity of the intervention (randomization, inclusion of a control condition, and adequate power); (2) incorporating smoking cessation intervention “best practices” (objective verification of smoking status, NRT, and long-term followup); and (3) systematically tailoring the ALA-FFS intervention modules (focus group research to guide development of peripheral, evidential, linguistic, sociocultural, and constituent tailoring strategies). A research study such as this would allow for a more rigorous test of the added benefits of culturally tailoring the ALA-FFS program for LGBT smokers.

## Figures and Tables

**Table 1:** Demographic, attitudinal, and smoking characteristics by treatment program offered.

Variable	Total (*N* = 198)	CIQ (*n* = 105)	BTQ (*n* = 33)	PIO (*n* = 60)	
Demographic characteristics	Mean (SD)/percent %				*p* values

Age	40.48 (9.22)	41.50 (8.83)	38.00 (8.58)	40.31 (9.99)	N/S
Race					<0.05
White	70.43	65.59	60.61	83.33	
Non-White	29.57	34.41	39.39	16.67	
Gender					<0.05
Male	60.51	76.70	0.00	66.10	
Female	35.38	17.48	93.94	33.90	
Transgender	4.10	5.83	6.06	0.00	
Education					N/S
High school or lower	11.68	15.38	6.06	8.33	
Some college	29.95	31.73	27.27	28.33	
College or above	58.38	52.88	66.67	63.33	
Employment status					<0.05
Full time	59.18	49.04	75.76	67.80	
Part time	6.12	7.69	0.00	6.78	
Unemployed	34.69	43.27	24.24	25.42	
Annual income					<0.05
$0–10,999	24.35	33.01	9.09	17.54	
$11–20,999	12.44	15.53	3.03	12.28	
$21–30,999	12.95	14.56	6.06	14.04	
$31–49,999	22.28	21.36	36.36	15.79	
$50,000 and over	27.98	15.53	45.45	40.35	
Sexual orientation					N/S
Homosexual	75.51	76.92	71.88	75.00	
Bisexual	10.71	8.65	12.50	13.33	
Others	13.78	14.42	15.63	11.67	
Smoking characteristics					
How much do you want to quit?					N/S
Definitely not	1.55	1.90	0.00	1.79	
Probably not	0.00	0.00	0.00	0.00	
Not sure	3.61	3.81	6.06	1.79	
Probably yes	32.47	33.33	30.30	32.14	
Definitely yes	62.37	60.95	63.64	64.29	
How important to quit?	4.34 (0.77)	4.23 (0.78)	4.30 (0.81)	4.57 (0.68)	<0.05
Not at all	0.00	0.00	0.00	0.00	
A little	1.55	0.95	6.06	0.00	
Moderately	13.40	18.10	3.03	10.71	
Very	34.54	38.10	45.45	21.43	
Extremely	50.52	42.86	45.45	67.86	
Do you intend to quit?					<0.05
Definitely not	0.00	0.00	0.00	0.00	
Probably not	0.00	0.00	0.00	0.00	
Not sure	9.79	12.38	9.09	5.36	
Probably yes	32.99	40.95	18.18	26.79	
Definitely yes	57.22	46.67	72.73	67.86	
How confident to quit?	3.36 (0.99)	3.33 (0.99)	3.55 (0.90)	3.30 (1.06)	N/S
Not at all	2.58	1.90	3.03	3.57	
A little	15.98	17.14	9.09	17.86	
Moderately	38.14	40.95	27.27	39.29	
Very	29.38	25.71	51.52	23.21	
Extremely	13.92	14.29	9.09	16.07	
Nicotine dependence	4.18 (2.49)	4.64 (2.45)	3.87 (2.58)	3.30 (2.29)	<0.05
Low	37.72	28.87	46.67	52.50	
Moderate	44.31	50.52	36.67	35.00	
High	17.96	20.62	16.67	12.50	
Nicotine replacement therapy					<0.05
Yes	24.24	27.62	36.36	11.67	
No	75.76	72.38	63.64	88.33	
Smoking cession outcomes					
Number of sessions attended	5.37 (2.03)	6.19 (1.74)	6.55 (0.89)	3.48 (1.47)	<0.05
Program completing					<0.05
≥75%	42.42	52.38	54.55	31.67	
<75%	57.58	47.62	45.45	68.33	
Quit rates (ITT at 1 month)					N/S
Yes	32.32	39.05	27.27	23.33	
No	67.68	60.95	72.73	76.67	

CIQ: Call It Quits; BTQ: Bitch To Quit; PIO: Put It Out; SD: standard deviation; N/S: not significant; ITT: intent-to-treat analyses.

**Table 2:** Multivariate analysis of factors associated with quit-smoking status (*N* = 121).

Variable	Odds ratio	95% CI	*p* values
Nicotine dependence	0.73	0.57–0.94	0.01
Nicotine replacement therapy	4.24	1.24–14.50	0.02
Number of sessions attended	2.45	1.45–4.15	<0.001

Race, gender, education, sexual orientation, attitudes about quitting smoking (want to quit, important to quit, intend to quit, and confidence to quit), and treatment program were adjusted for in the model.

**Table 3:** Multivariate analysis of factors associated with session attendance rates (*N* = 121).

Variable	Beta coefficient	S.E.	*p* values
Education (reference: college or above)			
Some college	−0.10	0.04	0.03
Nicotine replacement therapy (reference: no)			
Yes	0.10	0.04	0.01

Race, gender, sexual orientation, attitudes about quitting smoking (want to quit, important to quit, intend to quit, and confidence to quit), nicotine dependence, and treatment program were adjusted for in the model. Only significant factors are shown in the table.
